# Bridging the gap – how to walk the talk on supporting early career researchers

**DOI:** 10.12688/openreseurope.15872.1

**Published:** 2023-05-04

**Authors:** Daniel Pizzolato, Andrea Reyes Elizondo, Noémi Aubert Bonn, Borana Taraj, Rea Roje, Teodora Konach

**Affiliations:** 1 KU Leuven, Leuven, Flanders, Belgium; 2 European Network of Research Ethics Committees, Bonn, Germany; 3 Leiden University, Leiden, The Netherlands; 4 VU University Amsterdam, Amsterdam, The Netherlands; 5 Hasselt University, Hasselt, Flanders, Belgium; 6 European Association of Research Managers and Administrators, Brussels, Belgium; 7 University of Split School of Medicine, Split, Croatia; 8 Austrian Agency for Research Integrity, Vienna, Austria

**Keywords:** Early career researchers, career support, capacity building

## Abstract

Early career researchers (ECRs) play a crucial role in European and nationally funded research projects. They are at the forefront of planning, conducting, analysing, and reporting research. As part of the SOPs4RI project funded by the European Commission, we, as ECRs and members of this project’s consortium, were given the opportunity to reflect on our role, obstacles, and possible opportunities that we experienced. Although several steps have been already taken to support early career researchers, more concrete actions have to be pursued.

In our opinion, the EC should take the lead and serve as a global frontrunner (taken as exemplary also by national funding agencies) in implementing initiatives to support early career researchers during their research trajectory. We opine that the European Commission should explicitly (i) *require the creation of a support system in which early career researchers will be able to build new skills and capacity,* (ii) *encourage and facilitate more involvement of early career researchers in decision-making roles of EC-funded projects,* and (iii) *provide resources to support career continuity between fixed-term contracts*. The suggested actions can help early career researchers build competencies and expertise to establish stability and continuity within the research environment or to embrace and excel in careers outside academia.

## Disclaimer

The views expressed in this article are those of the authors. Publication in Open Research Europe does not imply endorsement of the European Commission.

Early career researchers (ECRs) play a crucial role in European and nationally funded research projects. They are at the forefront of planning, conducting, analysing, and reporting research. As part of the SOPs4RI project funded by the European Commission (EC), we, as ECRs and members of this project’s consortium, were given the opportunity to reflect on our role, obstacles, and possible opportunities that we experienced. We use the term *early career researchers* to include the broad range of research-enabling professionals in the early stages of their careers, regardless of age. This includes early career researchers (e.g., PhD students, junior postdoctoral researchers and early career professionals outside of academia such as research administrators and project managers) who are involved in funded research activities and anyone else contributing to research activities during the early stages of their career or career track. ECRs often face a challenging career transition, especially in the post-PhD period ^1^. Post-PhD transition is often characterized by precarious contracts, limited access to career development and funding, lack of mentorship, and other challenges inherent to the transition towards more independent careers.

Despite the fact that a road map to improve and support the lives of ECRs in Europe has already been drawn ^2^, further concrete actions are needed to support them in more substantial ways. Given its important role in numerous ECR careers, the European Commission has a responsibility to take the lead on this and require grant beneficiaries to be proactive in supporting ECRs within ongoing projects and in their career continuity.

Simultaneously with the presentation of the manifesto “Europe supports early research careers and stimulating research workplaces” at the 4th Gago Conference on European Science Policy in Brussels in June 2022, we reflected on how the European and national funding agencies can concretely support ECRs during their academic trajectory and in building future opportunities. In September 2022, a Manifesto for Early Career Researchers was launched to request broad and robust support to early career academics and scholars. The Manifesto has already been endorsed by many European and national organisations. This is a good first step; however, more specific actions can be taken by the EC when granting research projects and consortia. In this commentary, we present some of these reflections and provide specific recommendations to improve the conditions of ECRs.

The first action to be taken at several levels is to sensitise the research environment to the fragile, unstable, and unfavourable professional and psychological situation in which ECRs work. Recent initiatives, such as the Research Mental Health Observatory (REMO) Cost Action, the Danish campaign "Please do not steal my work", or the French collective Camille Noûs, raise awareness of some of the difficulties ECRs face. These recent initiatives are a good start and they suggest a shared understanding of the problems, but more needs to be done to bring this awareness to the different layers of the research ecosystem and encourage action by incorporating the topic to conversations at all levels, within departments, funding divisions, projects, etc.

Proactive support for ECRs can be provided at different levels, including 1) by European and national research funding organisations, 2) by research institutions, and 3) by senior colleagues who are involved in the same research project or have been involved in previous ones. Actions these stakeholders can take may include allocating budget to be used after the last deliverable of a project to enable ECRs to complete publications and/or prepare for new academic roles, and inviting ECRs to actively participate in the preparation of research proposals long before the end of the running project to increase the chances of a continuing career.

In our opinion, the EC should take the lead and serve as a global frontrunner (taken as exemplary also by national funding agencies) in implementing initiatives to support ECRs during their research trajectory within EC-funded projects and to promote career stability and continuity. We opine that the EC should explicitly (i) *require the creation of a support system in which ECRs will be able to build new skills and capacity,* (ii) *encourage and facilitate more involvement of ECRs in decision-making roles of EC projects,* and (iii) *provide resources to support career continuity between fixed-term contracts*. As in the case of explicit initiatives in support of open access, the EC should take the lead in supporting ECRs. We propose a list of six actions that can serve as a starting point to further develop initiatives in support of ECRs ( Box 1).

**Box 1. The proposed actions EC should take to support early career researchers**

**Table:** 

**Support and capacity building** Action 1: Ombuds system and confidential advisors Action 2: Research integrity and research ethics advisory system Action 3: Capacity-building budget for ECRs **Involvement in decision-making** Action 4: ECR inclusion in decision-making and leadership roles **Career continuity** Action 5: Bridge careers after project completion Action 6: ECR network and community platform

***Action 1: Ombuds system and confidential advisors (Support and capacity-building)***

In funding research consortia or single-beneficiary grants, the EC could require funded institutions or consortia to have an ombuds system in place to handle issues that might arise in relation to the psychological well-being and workplace environment of ECRs. The EC could require project leaders to assign a confidential advisor who is aware of the resources and contact persons and who is knowledgeable with regards to Diversity, Equity, and Inclusion (DEI) principles and is able to direct the consortium to appropriate DEI training. DEI training and policies should be available in the different institutions involved in the project. In fact, in addition to the issues that most ECRs face in normal research settings ^3, 4^, some ECRs may experience further difficulties and power imbalance because of different characteristics such as gender identity, dis/ability ^5^, nationality, and socio-economic or cultural background, which often vary between the different hierarchical levels within academia. It is important that consortia and institutions where the funded researchers are based have measures in place such as experts and policies to address these inequalities. The SOPs4RI project co-created guidelines for research institutions on diversity and inclusion which may provide tips on how to address these issues.

***Action 2: Research integrity and research ethics advisory system (Support and capacity-building)***

In funding research consortia or single-beneficiary grants, the EC could require that a research integrity and research ethics advisory system is in place to support and advise project members at all career stages in terms of responsible research practices and conduct. Scientific misconduct and questionable research practices are not just due to deliberate misbehaviour or sloppy practices. They are also caused by a lack of knowledge, inadequate education, and limited awareness about the possible consequences of questionable practices. Excessive expectations, unreasonable time pressures, and peer pressure where seniors engage, in questionable research practises can also come into play. Having research integrity and ethics advisors, within or outside the consortium, can help ECRs and seniors to clarify uncertainties, fill possible lacunas of knowledge and competencies, and reflect on what constitutes responsible research practices.

***Action 3: Capacity-building budget for ECRs (Support and capacity-building)***

In funding research consortia or single-beneficiary grants, the EC could support and encourage applicants to apply for a capacity-building budget for ECRs. When funding research projects, the EC could encourage and support consortia that apply for extra budget to focus on providing training sessions and building skills, including skills that extend beyond the needs of the project and that are useful for careers outside academia. Encouraging applicants to include this extra budget in their funding applications would allow ECRs to build extra competencies, skills, and expertise. The SOPs4RI project co-created guidelines for institutions on education and training which may be useful in identifying the areas and strategies to address the needs of ECRs.

***Action 4: ECR inclusion in decision-making process (Involvement in decision-making)***

In funding research consortia or single-beneficiary grants, the EC could require consortia to involve ECRs in the decision-making process throughout the research project. This could include, for example, involving ECRs in strategic decisions for the preparation, completion and success of the project, requiring consortia to have ECRs in leadership roles (for instance as work-package co-leaders), and participation of ECRs in the Advisory Board or Executive Committees. Complementing the added perspective that this would provide, involving ECRs in core decision-making processes can help them acquire experience and expertise in how to manage important issues related to the research environment and research process, while offering them a safe environment in which they can prepare for future leadership roles. Moreover, the EC could encourage the involvement and recognition of ECRs in the process of writing research proposals. When evaluating research proposals, the EC should require consortia to fully involve ECRs in writing the grant proposal and to clearly detail how the consortium or single-beneficiary grant intends to involve ECRs during the lifetime of the project. The involvement of ECRs in the phase of writing a new grant proposal could increase opportunities of career continuity. To capture this, the EC could expand the ‘gender perspective’ that is currently requested in proposals to a more general ‘Commitment to diversity statement’, which explicitly includes gender, diversity (e.g., cultural, racial gender, physical and diversity in interests), inclusion, together with early career researcher perspectives.

***Action 5: Bridge careers after project completion (Career continuity)***

In funding research consortia or single-beneficiary grants, the EC could foresee additional funding to bridge precarious early careers after project completion. Several consortia already encourage ECRs to apply for future funding when approaching project completion, but this often happens without formal funding and outside working hours, leading to exhaustion and frustration. When funding research projects, the EC could guarantee extra funding to bridge transition periods inherent to early careers. This could involve additional months of funding for ECRs after the completion of the project during which ECRs could complete publications, build additional competencies, and apply for future funding opportunities. This has already been well implemented in the U.S. and allows ECRs to fill the gap between fellowships.

***Action 6: ECR network and community platform (Career continuity)***

The EC could promote and support the creation and consolidation of a network and community platform to support ECR career stability and continuity. The community platform can serve as a contact point between ERCs with specific expertise and project leaders of starting EC-funded projects. As in the case of the ENERI e-community, the database could be integrated into SINAPSE, a free public service provided by the EC, or in the new EC-funded online platform, the Embassy of Good Science. In addition, the community platform could offer a springboard for networking and ERCs to exchange ideas, share resources, and initiative activities, not only among themselves but with more seasoned researchers. 

To conclude, we believe that EC-funded projects offer an ideal platform to support ECRs as they embark on their academic career or future professional trajectory. The suggested actions can help them build competencies and expertise to establish stability and continuity within the research environment or to embrace and excel in careers outside academia. We hope these suggestions might inspire the EC to assume a leadership role in supporting ECRs, while encouraging and incentivising research institutions and research funders to join this important effort. In addition, we think that these suggestions can be framed in the context of existing EU policies, such as initiatives for reforming research assessment, gender equality in research, and research integrity, among others designed to enhance the research endeavour.

## Ethics and consent

Ethical approval and consent were not required.

## Data Availability

No data are associated with this article.
