# Supplementary material for: Bridging the gap – how to walk the talk on supporting early career researchers
Source: Open Res Eur. 2023 May 4;3:75. [Version 1] doi: 10.12688/openreseurope.15872.1 (PMC10509601; doi:10.12688/openreseurope.15872.1)
Supplement: Peer review history

# Reviewer report: Reviewer response for version 1

**DOI:** 10.21956/openreseurope.17145.r34844

**Published:** 2023-09-26

**Authors:** Yoseph Araya

**Affiliations:** 1 The Open University, Milton Keynes, England, UK

This is a neatly communicated letter addressing the important challenge facing early career researchers (ECRs), a secure transition to academic and research career. It is built on the reflections of researchers from a European Commission (EC) funded project, undertaken across various European countries. The outcomes are largely targeted at the EC level and how it can play a role in ensuring a career path for ECRs. Citing various examples, of which there are plenty, it highlights the precarious nature of transition faced by ECR’s as a result of their limited contracts, in their most vulnerable career stage. They are still learning research skills and still building experience forging their academic career, when they eventually face the abrupt step of ensuring the next long-term contract. Too often they are unprepared to obtain their own grant, especially with the competition for limited opportunities. This inevitably has, and is highlighted by the authors, apart from financial, mental wellbeing consequences. This is unfortunately a reality shared globally, with similar cases for example in the UK and USA. The letter is aimed at the EC, a major funder, to act as a leader in ensuring ECR research trajectory continues. The proposal suggested rightly focuses on support at the beginning, whilst still in the early career project, and once near the finish. A few points and suggestions the authors could elaborate or discuss are presented as follows.

 Considering the first of the three stages suggested, support in the early stage of the career is crucial in building the skills and confidence needed in a research career. The focus on building a formal structure on training and mentorship is well made. There are examples of this being undertaken by multiple institutions elsewhere too. For e.g. UK Research and Innovation body (UKRI) has recently introduced Doctoral Training Partnerships ( https://www.ukri.org/what-we-do/developing-people-and-skills/esrc/doctoral-training-partnerships/), which not only train ECR’s in various skills from the perspective of a broader inter-university and research partner collaboration. The key with such approach is having a coordinating body. Although the suggestion of assigning confidential advisors (‘Ombuds’ or mentors is suggested in the letter, the need for a formal structure within the projects would be necessary to realise the desired support outcomes. It is crucial who and where the advisor(s) sit and how they operate. It is also important to specify how the Ombuds will collaborate within projects. The letter rightly emphasises on Ombuds being familiar with diversity and inclusion principles. This is especially even more challenging across a wide network of different countries as would be anticipated in EC countries. Ensuring a ring-fenced budget for capacity building is an excellent idea, but it is crucial national governments and institutions, ensure existence and continuity of such advisory and training work. So this should be potentially a requirement before EC grants are awarded. A suggestion on how such structure can be created and maintained hence would be a good discussion point.

 Considering support whilst during the grant in the form of inclusion in decision-making and leadership is rightly made. However, this probably is broad issue, which will inevitably deal with research culture and ethics. Recently a lot has been discussed including an excellent paper by ( Maestre et al, 2019). However, to ensure practicality, it might have to be supported via individual institutions and nations as well. For example, a wider recognition of good culture could be considered such as a charter or concordat which institutions sign up (e.g. UK’s concordat for research integrity or the Athena Swan Charter). Recognition for institutions that demonstrate good support on such initiatives, could then be used as an additional criteria by the EC when considering funding bids. The key thing is good culture of supporting ECR’s should be practically assessed and become a motivating factor for institutions bidding for EC funds.

 Considering the latter part of an ECR’s involvement in a project and the next step, a network of ECR’s will be crucial and is well pointed at. Good examples are provided and may be stronger case could be made in the letter, how this should be well-established and embedded within projects. Discussion could be made if there a case to be made for an independent EC funded body working across projects.

 Overall an engaging and well written letter, with clear ideas on how to support ECR’s. It will be rewarding to have EC and national bodies take steps to ensure the future of researchers.

Where applicable, are recommendations and next steps explained clearly for others to follow? (Please consider whether others in the research community would be able to implement guidelines or recommendations and/or constructively engage in the debate)

Yes

Does the article adequately reference differing views and opinions?

Yes

Are all factual statements correct, and are statements and arguments made adequately supported by citations?

Yes

Is the rationale for the Open Letter provided in sufficient detail? (Please consider whether existing challenges in the field are outlined clearly and whether the purpose of the letter is explained)

Yes

Is the Open Letter written in accessible language? (Please consider whether all subject-specific terms, concepts and abbreviations are explained)

Yes

Reviewer Expertise:

NA

I confirm that I have read this submission and believe that I have an appropriate level of expertise to confirm that it is of an acceptable scientific standard.

# Reviewer report: Reviewer response for version 1

**DOI:** 10.21956/openreseurope.17145.r34845

**Published:** 2023-09-19

**Authors:** Victoria L. Hewitt

**Affiliations:** 1 The University of Auckland, Auckland, Auckland, New Zealand

The Open letter makes clear, actionable policy suggestions directed at the EC that are also more broadly applicable to research funding bodies worldwide. Making adequate provisions for career progression and protection from exploitative employment practices a requirement of external funding would provide a strong incentive to employers of ECRs to improve their working conditions and thereby empower a vital part of the research sector. These concrete suggestions tied to funding, but in most cases not dependent on additional funding being sourced or structures being developed, offer clearer steps forward than the 2022 Manifesto by the Initiative for Science in Europe (ISE) and the 2005 Euraxess Charter. Citing the charter and noting the limited progress since 2005 would also strengthen the author’s argument that more concrete steps are necessary to achieve a culture change.

 The introduction clearly explains the aims of the letter, but it would be helpful for many readers to have a very brief introduction to the SOPs4RI program to ensure the context of the text can be understood without following further links. The authors acknowledge that commitment to adopting the recommendations of the Manifesto at an institutional level are a good first step but could add that this helps ensure many improvements are accessible to all ECRs not just those on EC funded projects.

 Regarding the advisors and additional capacity training referenced in Actions 2 and 3, the cost of funding and finding suitable people to fill these roles may be difficult for some smaller institutions. Perhaps alternatives in the form of a small fund from the grant to gain relevant but otherwise unavailable training for one of the applicants could also help capacity building and spread best practice in these areas.

 Action 4 suggests naming and including ECRs in the early stages of the grant application process and on the evaluation side. These are relatively easy steps that simply allow ECRs “in the room” but are potentially transformational for ECRs whose experience may otherwise be completely dependent on their supervisors to provide these opportunities (and might otherwise be missed because of time pressures and lack of awareness around training of ECRs).

 The bridging funding suggestions in Action 5 also have the potential to alter career paths particularly for ECRs in systems where less institutional or national funds are available and in the scale of EC grants would not have huge impact on costs. In my experience ensuring the availability of such funding reaches the appropriate people appears to be a limiting factor and indicating as such in any guidance around documentation for applications would be prudent.

 The ECR network suggested in Action 6, while appealing, is (in agreement with the other reviewer, Brian Cahill) hard to implement. Without significant administrative support and institutional “champions” leading the way such networks are very hard to sustain. A more targeted network as suggested (the ENERI e-community) is one avenue that appears to help ensure continuity. Having seen the decline of a once very useful academic twitter and more discipline specific society and institutional initiatives I feel this action is less easily implemented than the rest. Perhaps this advice could be amended to include advice that EC funding applications note concrete ways ECRs involved will be encouraged make connections via access existing networks (eg Professional societies, inter-institutional and nationally funded network programs/exchanges).

 Beyond inclusion of a very brief introduction to SOPsRI and the addition an “Introduction” heading to the text beginning after the disclaimer my other comments are simply suggestions and I recommend publication of this Letter.

Where applicable, are recommendations and next steps explained clearly for others to follow? (Please consider whether others in the research community would be able to implement guidelines or recommendations and/or constructively engage in the debate)

Yes

Does the article adequately reference differing views and opinions?

Yes

Are all factual statements correct, and are statements and arguments made adequately supported by citations?

Yes

Is the rationale for the Open Letter provided in sufficient detail? (Please consider whether existing challenges in the field are outlined clearly and whether the purpose of the letter is explained)

Yes

Is the Open Letter written in accessible language? (Please consider whether all subject-specific terms, concepts and abbreviations are explained)

Yes

Reviewer Expertise:

I am a Researcher Developer and have extensive experience as an ECR across 3 continents and 5 institutions and have been involved in supporting ECRs for many years while also performing research.

I confirm that I have read this submission and believe that I have an appropriate level of expertise to confirm that it is of an acceptable scientific standard.

# Reviewer report: Reviewer response for version 1

**DOI:** 10.21956/openreseurope.17145.r31441

**Published:** 2023-05-22

**Authors:** Brian P. Cahill

**Affiliations:** 1 Leibniz Information Centre for Science and Technology, Hannover, Germany

This a well written and timely call for greater involvement of researchers in framing the future of the European Research Area and in creating structures that better support researchers.

 One weakness of this paper is the lack of assessment of the current frameworks that address such issues and the involvement or lack thereof of ECRs in their administration. In 2005, the European Commission published the European Charter for Researchers and a Code of Conduct for the Recruitment of Researchers ^1^^ ^( https://euraxess.ec.europa.eu/sites/default/files/am509774cee_en_e4.pdf). Ten years after its publication, one of the organisations that actively lobbied for the Charter and Code to be adopted, EuroScience, published a very lukewarm appraisal of the "success" of the Charter and Code ( https://www.euroscientist.com/does-the-european-researchers-charter-deserve-its-birthday-cake/) ^2^. The administration of the Charter and Code has been largely at a level that ECRs have no input at a national or institutional level. In some countries (for instance, Germany), very few institutions have endorsed the Charter and Code.

 The Charter and Code appears to be being revised at the moment through a consultative process that I understand involves a small number of researchers and extensive representation from national governments and other stakeholders.  Researchers have some representation but people working in relevant roles as ombuds, research integrity officers, researcher career developers etc. are almost completely excluded. This compares very poorly with the revision of the Concordat to Support the Career Development of Researchers ^3^ (the UK equivalent) published in 2019 that was led by Vitae and that included feedback from all relevant stakeholder groups through an open call for contributors to a Concordat writing group.

 Many national funders (German Research Council DFG) allow a cost-neutral extension of the project beyond the originally-agreed project lifetime that often allows ECR contracts to be extended. This is not a usual feature of EC-funded projects that tend to impose fixed deadlines for project completion and use of budget. I agree that a dedicated budget to support ECRs in this way would be very useful. Particularly the 3-year funding for MSCA Doctoral Fellows causes a huge amount of mental stress for young researchers, who are required by the eligibility criteria to be internationally mobile and who often take longer than 4 years to complete their PhD. This is a structural issue within the conception of doctoral funding that places undue stress on ECRs and leads to reduced levels of completion of doctoral programmes.

 As regards Action 1, the Charter and Code already includes a recommendation to implement an ombudsman-type system within institutions and research funders. Nevertheless the devil is in the detail. It is accompanied by a qualification that "in compliance with national rules and regulations, appropriate procedures". The European Commission has no competence to enforce the non-binding recommendations within the Charter and Code. This remains the competence of relevant national bodies, such as the national legal system.

 As reagrds Action 2, there is a very variable coverage of training for research integrity throughout Europe. Many researchers (particularly more senior researchers) have never received research integrity training of any sort and have absorbed experience through a process of osmosis from their former supervisors, colleagues and general experience of working as an academic (I count myself among these individuals). In some countries (Germany), research integrity officers are rather confusingly called Ombuds, which confuses many researchers looking for advice on issues related to power abuse, bullying and harassment.

 The European Commission includes many of the Capacity Building measures mentioned under Action 3 in the Marie Sklodowska-Curie Actions, Erasmus Mundus and the EIT. A more general access to such training and network-building activities across all EU-funded programmes would be highly welcome. The Commission has recently funded the EURAXESS Hubs project ^4^ that aims to build support for researcher careers through the EURAXESS network. This network is useful but is more focused on supporting researcher mobility through an extensive network of international offices throughout Europe rather than an extensive network of researcher career developers. It is questionable how effective these sort of projects can be without directly engaging career developers at the institutional level.

 In general, the European Commission considers  academic researchers as being represented by their institutions.This is highly questionable. From a purely legal viewpoint, the beneficiary is the institution that communicates with the Research Executive Agency and employment-related issues are largely a matter for institutional Human Resources to deal with. The Commission set up the Human Resources Strategy for Researchers (HRS4R) as a type of accreditation process for the implementation of the Charter and Code. Adding ways to include involvement of ECRs in strategic decisions related to EU-funded projects does not play any role in HRS4R. In MSCA projects, the ECR and the supervisor must jointly draft a Career Development Plan for the ECR but more direct involvement of ECRs in higher-level management is not foreseen. Even the career development plan is not a requirement of other EC-funded programmes.

 As regards Action 5, the Commission previously had a relatively small-valued grants for Career Reintegration of MSCA fellows that supported the fellows to move institution and retain a small amount of funding to support their continuing career development. I personally benefited from this programme. My understanding is that it was discontinued during FP7 due to the expense of awarding and evaluating many small-valued grants (that were of huge value to the careers of ECRs). Lack of flexibility at early-career stages can be very disruptive to the career development of researchers especially in combination with international mobility.

 As regards Action 6, the Commission set up exactly this sort of ECR network and community platform in 2012. It was called "Voice of Researchers" and its surviving footprint is limited to a Twitter presence ( https://twitter.com/Research_Voice). If activities of this sort are purely based on time-limited funding, a repetition of the demise of the Voice of Researchers portal is very likely. Also, it must be taken into account that a representative group of individuals without an independent status that is purely funded by the Commission with the aim of giving feedback to the Commission is something that might lead to other stakeholders dismissing the feedback of these ECRs as being overly biased towards Commission policy positions.

 On a final point that might be considered biased (Conflict of Interest declaration: I am grant manager of the ReMO COST Action on Researcher Mental Health), the ReMO COST Action is not purely focussed on Raising Awareness. Raising awareness of  researcher mental health is one important objective of several mentioned in the *Researcher Mental Health and Well-being Manifesto* ^5^ *^^*. ReMO is also running a Europe-wide survey, drafting national policy briefs and supporting the collection of evidence on the effectiveness of interventions to support researcher mental health.

Where applicable, are recommendations and next steps explained clearly for others to follow? (Please consider whether others in the research community would be able to implement guidelines or recommendations and/or constructively engage in the debate)

Yes

Does the article adequately reference differing views and opinions?

Yes

Are all factual statements correct, and are statements and arguments made adequately supported by citations?

Yes

Is the rationale for the Open Letter provided in sufficient detail? (Please consider whether existing challenges in the field are outlined clearly and whether the purpose of the letter is explained)

Yes

Is the Open Letter written in accessible language? (Please consider whether all subject-specific terms, concepts and abbreviations are explained)

Yes

Reviewer Expertise:

Interdisciplinary; Researcher Career Development; Researcher Mental Health; Research Policy; Microfluidics; Colloid and Interface Science; Biomedical Engineering; Sensors and Actuators;

I confirm that I have read this submission and believe that I have an appropriate level of expertise to confirm that it is of an acceptable scientific standard.

## References

[1] : European Charter for Researchers and a Code of Conduct for the Recruitment of Researchers.2005; Reference source

[2] : Does the European Researchers Charter deserve its birthday cake?. *EuroScientist* .2015; Reference source

[3] Concordat to Support the Career Development of Researchers. *Vitae* .2019; Reference source

[4] : Researcher Mental Health and Well-being Manifesto. *Zenodo* .2021; 10.5281/zenodo.5559806 10.5281/zenodo.5559806

[5] Piloting EURAXESS talent hubs to support researchers’ careers. 10.3030/101035541 10.3030/101035541
